# Efficacy and safety of 3D printing-assisted percutaneous nephrolithotomy in complex renal calculi

**DOI:** 10.1038/s41598-021-03851-2

**Published:** 2022-01-10

**Authors:** Dong Cui, Fengqi Yan, JiangPu Yi, Dali He, Yichen Zhang, Zekai Zhang, Yuntao Chen, Yong Jiao, Bo Zhang

**Affiliations:** 1 Department of Urology, Tangdu Hospital, The Air Force Military Medical University, Xi’an, Shaanxi China; 2 Department of Ultrasound Diagnostic, Tangdu Hospital, The Air Force Military Medical University, Xi’an, Shaanxi China

**Keywords:** Urology, Urogenital diseases, Urinary tract obstruction

## Abstract

This study evaluated the efficacy and safety of 3D printing technology combined with percutaneous nephrolithotomy in the treatment of complex renal calculi. Ninety patients with complex renal calculi were randomly divided into a 3D printing group (45 patients) and a control group (45 patients). In the 3D printing group, a patient-specific 1:1 3D printing model was established based on the patient's thin-layer CT scanning data. A 3D printing model was used for preoperative communication between doctors and patients. Preoperative puncture training, channel design, residual stone prediction, and percutaneous nephrolithotomy were performed under the guidance of a 3D printing model and B-ultrasound. The control group was treated with the conventional B-ultrasound-guided puncture method. Results suggest that there was a statistically significant difference between the two groups (P < 0.05). The overall score of the doctor-patient communication objects in the 3D printing group was 19.32 ± 1.57 points, and in the control group, it was 14.51 ± 2.13 points. The operation time of the 3D printing group was 103.21 ± 13.49 min, and that of the control group was 126.12 ± 25.87 min. The calculi clearance rate of the 3D printing group was 96%, while that of the control group was 80%. The incidence of postoperative complications was 6.67% in the 3D printing group and 22.22% in the control group. Compared with traditional percutaneous nephrolithotomy, 3D printing technology combined with percutaneous nephrolithotomy can significantly enhance the effectiveness of doctor–patient communication, shorten operation time, reduce operation bleeding, improve the stone clearance rate, reduce the incidence of complications and shorten the length of hospital stay. The proposed method is thus a safe and effective method to treat complex renal calculi.

## Introduction

Nephrolithiasis is a common disease affecting urinary tract, which seriously affects the renal function of patients. In complex renal calculi, such as multiple stones, horseshoe kidneys, staghorn calculi, sponge kidneys, etc^1–3^, calculi are usually larger than 2.5 cm. Percutaneous nephrolithotomy is commonly used in the clinical treatment of complex renal calculi, but there is also a high risk of complications and a high incidence of bleeding and infection^4^.

3D printing is a new technology that has seen rapid development in recent years that is based on digital model files and uses adhesive materials to print layer by layer according to computer-aided drawings, thus transforming the data on the computer into real objects. Currently, 3D printing has been widely used in orthopedics, cardiac surgery, neurosurgery and other medical fields^5^ for preoperative communication, preoperative planning and training, and to predict surgical effects^6^.

For simple renal calculi, compared with open operations, percutaneous nephroscopy has the advantages of minimum trauma, less bleeding and fewer complications^7^. Traditional B-ultrasound or X-ray guidance can be successfully completed, but for complex renal calculi, due to its complex operation and long operation time, the incidence of complications is high^8^. Some studies have investigated using 3D printing and percutaneous nephroscopy to treat simple^9, 10^ and staghorn stones^11^; however, no prospective randomized controlled study has been performed on this combination’s application to complex renal calculi. Therefore, we evaluate the therapeutic effect of 3D printing combined with percutaneous nephroscopy in the treatment of complex renal calculi.

## Materials and methods

### Patient selection

Patients with complex kidney stones who met the indications for percutaneous nephrolithotomy in a hospital from May 2017 to May 2021 were selected. Renal calculi was assess by CT, the definition of complex renal calculi was “CT examination of stone diameter greater than 2.5 cm, multiple stone, staghorn calculi, horseshoe kidney, and medullary sponge kidney”. The inclusion criteria was CT examination of stone diameter greater than 2.5 cm, multiple stone, staghorn calculi, horseshoe kidney, medullary sponge kidney. And the exclusion criteria was CT examination of stone diameters less than 2.5 cm, single stones, ureteral stones, prior history of percutaneous nephroscopic surgery, abnormal coagulation and abnormal cardiopulmonary function or other surgical contraindications. A total of 121 patients were assessed for eligibility, and 31 patients were excluded, including 15 cases of a single stone, 6 cases of stone diameter less than 2.5 cm, 3 cases with a prior history of percutaneous nephroscopic surgery, 3 cases of abnormal coagulation and 4 cases of abnormal cardiopulmonary function. 90 patients met the inclusion criteria, including 61 male patients and 39 female patients. No patients had a definite contraindication to surgery. After obtaining informed consent from all patients, they were randomized into two groups using a computer-generated randomization schedule. The 3D printing group (*n* = 45) had a body mass index of 24.31 ± 3.42 kg/m^2^. The mean diameter of their kidney calculi was 3.81 ± 1.31 cm, while the control group (*n* = 45) had a body mass index of 23.13 ± 3.15 kg/m^2^. The mean diameter of their kidney calculi was 3.60 ± 1.35 cm.

### Establishing and application of the 3D printing model

**Figure 1:**
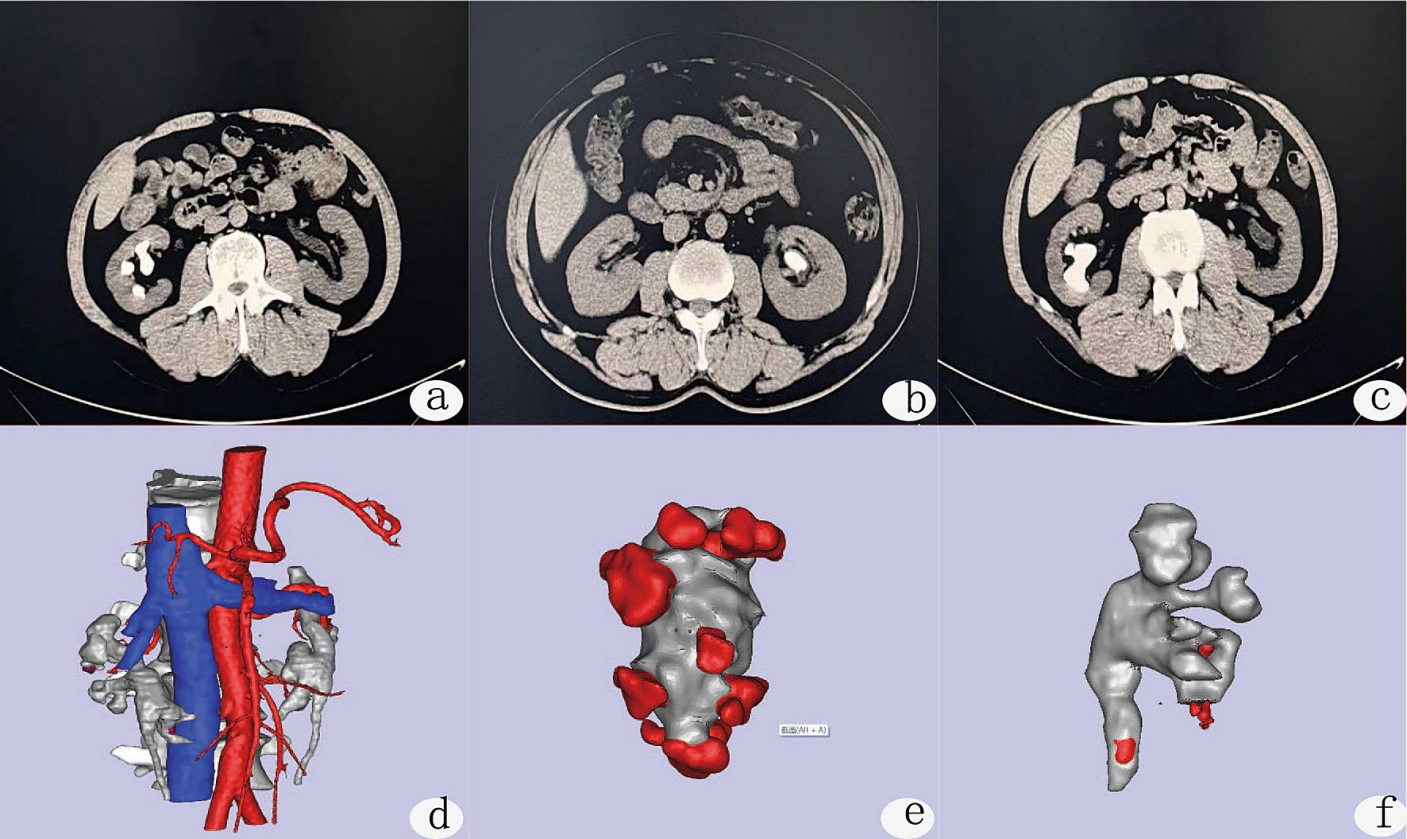
CT and three-dimensional reconstruction of different parts of the kidney. **(a–c)** Typical preoperative CT of the patients; **(d–f)** three-dimensional reconstruction images of different parts of the kidney. **(d)** Distribution of renal blood vessels, **(e)** renal pelvis and calculi, **(f)** three-dimensional reconstruction of the renal pelvis and the size and location of the calculus.

**Figure 2:**
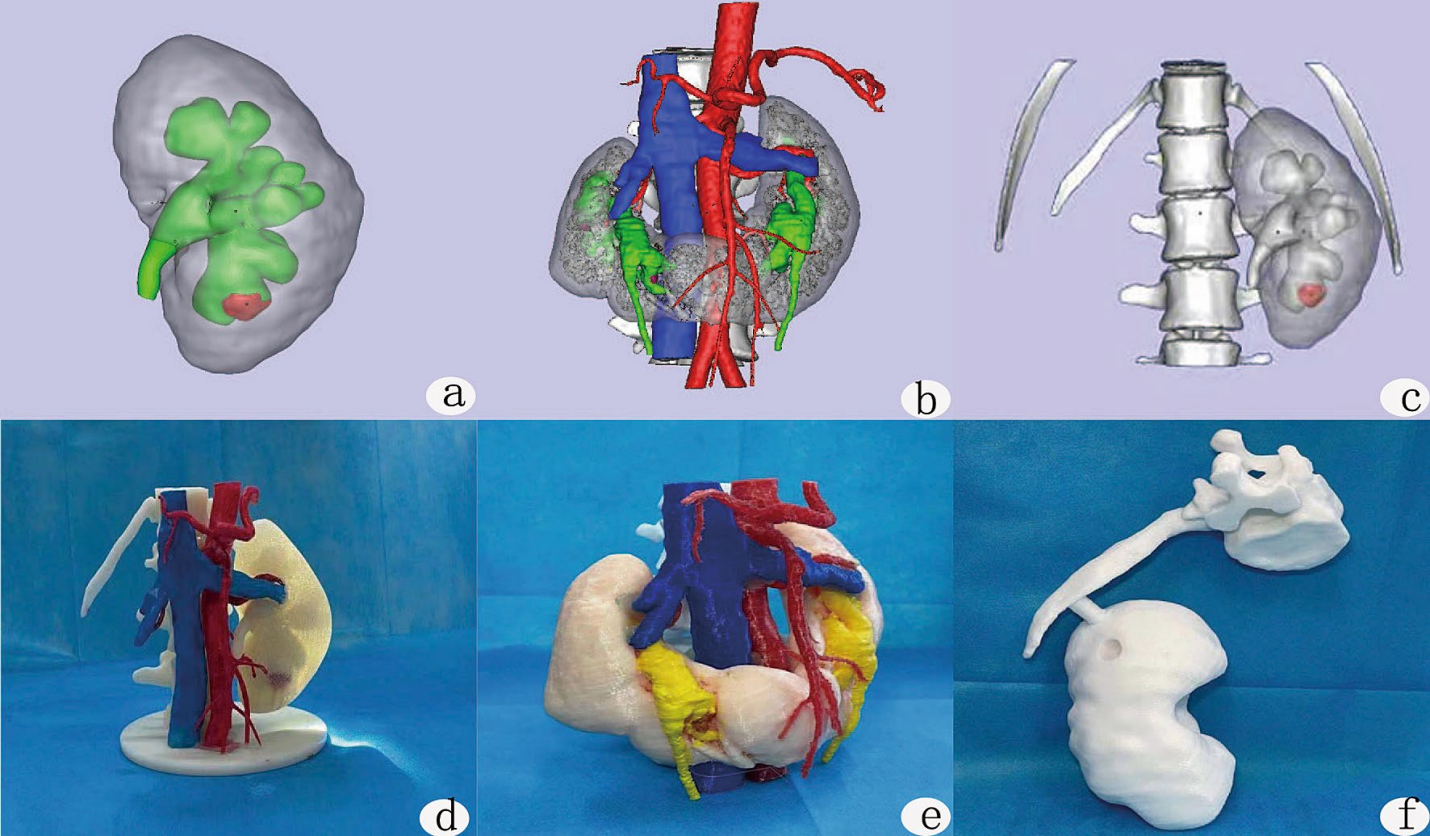
Renal 3D reconstruction and printing results. **(a)** Three dimensional reconstruction of intact kidney; **(b)** three dimensional reconstruction of kidney and blood vessels; **(c)** three dimensional reconstruction of kidney, spine and rib; **(d–f)** three dimensional printed kidney model.

CT-scan images were done in prone position. The thin-layer CT scan images of the patients' kidneys were extracted, (Fig. 1a–c) DICOM format files were extracted from the CT scans, and images were processed with Mimics 17.0 software (Materialise Inc., Leuven, Belgium. https://www.materialise.com). After using threshold selection, region growth, multilayer editing and modification techniques, three-dimensional reconstruction images of different parts of the kidney were obtained (Fig. 1d–f) and then combined into a complete kidney. (Fig. 2a,b) In addition, the 11th and 12th ribs were reconstructed together for smoothing, fine filling and other post processing (Fig. 2c). Finally, a standardized STL file for 3D printing was output. Using Mimics 17.0 software (Materialise Inc., Leuven, Belgium. https://www.materialise.com), we drew the puncture point and designed the best puncture channel, puncture depth and puncture track in the combined three-dimensional graphics of the kidney. A 3D printer (Shanghai liantai Technology Co., Ltd. rs4500) was then used to print the 3D model.

The 3D printed model was used to communicate with patients or guardians preoperatively to help patients or guardians fully understand the necessity of surgery, the surgical methods, procedures, expected results, operation risk and postoperative complications. Patients provided an overall score about how helpful the 3D printing model was for them to understand the surgical plan. Questionnaires were used with the patients or their authorized persons, and asked about the following topics: (1) the usefulness of the 3D printing model for understanding kidney calculi disease; (2) the usefulness of the 3D printing model for understanding kidney structure and calculi distribution; (3) the usefulness of the 3D printing model for understanding the operation risk and the operation method; and (4) their overall satisfaction with this communication conversation. Scores from low to high were 1–5, where 1 indicated useless/very dissatisfied, and 5 indicated very useful/very satisfied. These scores were used to evaluate the effect and satisfaction of preoperative communication.

Using the imaging data and 3D printing model, the surgeons planned the percutaneous nephrolithotomy channel, target renal calyx, puncture angle, predicted the residual stones, puncture depth and lithotripsy process (Fig. 2d–f). Through the 3D printed images of the patient's kidney and stones to establish the best gravel channel, which was the shortest distance between the skin and the target calyx, and will form an obtuse angle with the surrounding target renal calyces. This gravel channel will be convenient for treating the stones of multiple calyces. If the angle between the neck of the renal calices containing stones and the best gravel channel is less than 60 degrees, it indicates that the nephroscope will not be able to enter the renal calyces for laser lithotripsy, and the stones in the renal calyces will remain as residual stones. The intersection of the horizontal distance from the proposed puncture point to the posterior midline and the distance from the proposed puncture point to the 12 costal tips is the skin puncture point of the percutaneous nephroscope. By measuring the distance between the puncture point and the target calyx, we roughly knew the puncture depth. We record the distance between the optimal puncture point and the posterior midline, the distance between the optimal puncture point and the 12 costal tip, the relationship between the optimal puncture point and the 12 costal tip (the puncture point is located above or below the 12 costal tip), the angle of the gravel channel and the back plane, and the angle of the gravel channel and the posterior midline. We reproduced the puncture angle depth and puncture process on the 3D printed model and conducted puncture training.

### Surgical procedure

**Figure 3:**
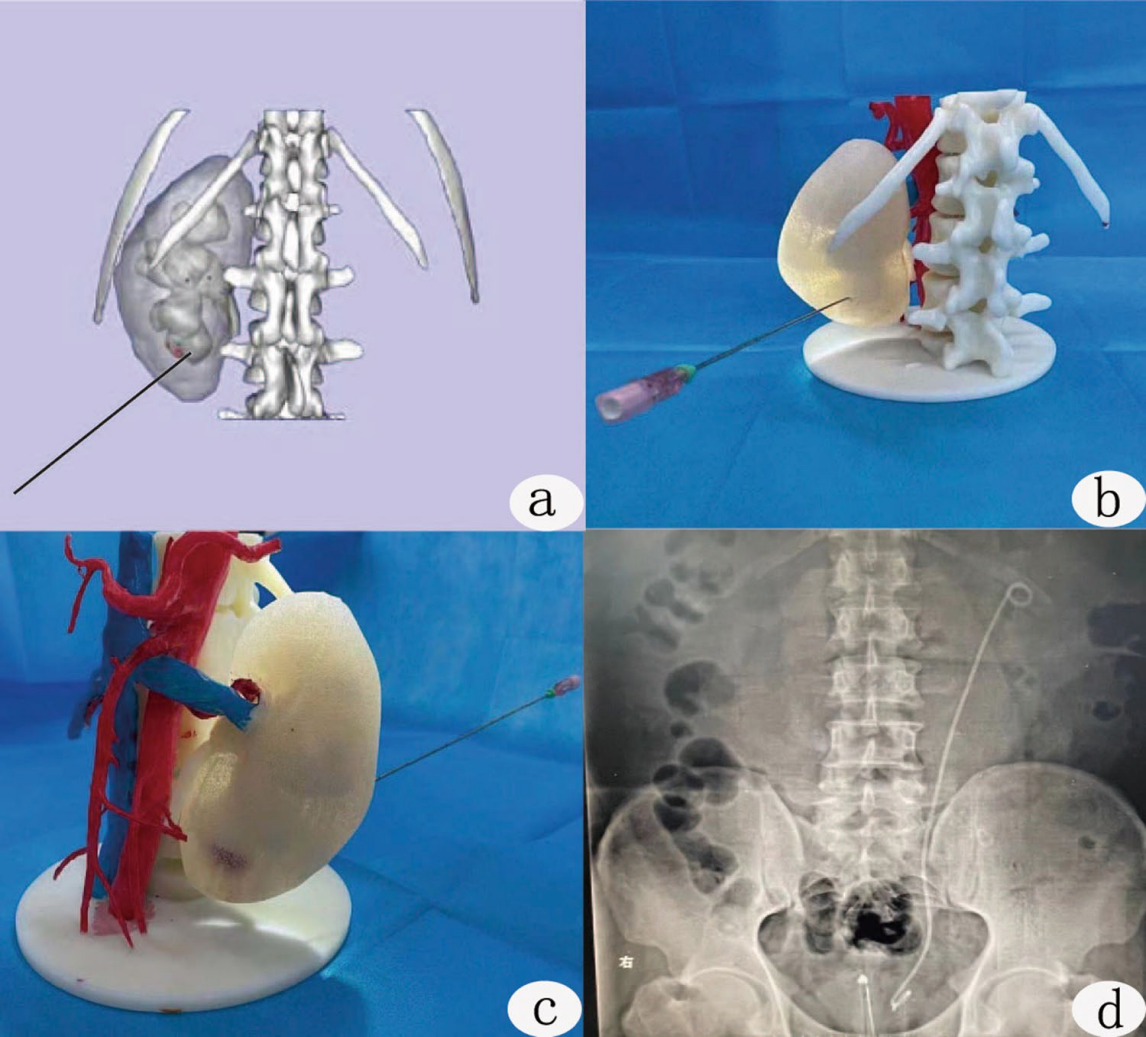
Puncture training and postoperative plain film. **(a)** Selection of puncture sites and calyces in a three-dimensional reconstruction image; **(b,c)** puncture training, target calyces, selection of puncture angle and estimation of puncture depth on 3D printed physical model. **(d)** Postoperative plain film.

One single surgeon with 2 years of experience in percutaneous nephrolithotripsy completed the operation. General anesthesia or epidural anesthesia was used. After successful anesthesia, the lithotomy position was taken. An F5 ureteral catheter was placed on the affected side by cystoscopy, and an F18 three-lumen catheter was placed to drain the bladder. When the patient was turned to the prone position, the appropriate calyces puncture was selected under the guidance of B-ultrasound. The 3D printing group used the channel designed on the 3D model before the operation (Fig. 3a–c), and the puncture point was generally located between the 11th intercostal or the 12th intercostal from the posterior axillary line to the subscapular angle line.

After successful puncture, a zebra urological guidewire was inserted along the puncture needle. The fascial dilator expands the puncture site from small to large to establish a suitable working channel. After the operation channel and calyces were examined via nephroscopy, the calculus was located, and holmium laser lithotripsy was performed. If necessary, multiple working channels were established to remove the calculus as much as possible. Then, an F6 double-J stent was inserted on the affected side, the ureteral catheter was removed, the nephrostomy tube was inserted on the affected side, and the operation was concluded. The plain film of the urinary system was rechecked 7 days after the operation (Fig. 3d). After operation, the operation time, puncture time, number of channels, the coincidence of puncture target calyces, blood loss, postoperative complications and calculus clearance rate of the two groups were recorded.

### Postoperative treatment

Patient temperature, urine culture and hematology examination were used to screen for postoperative infection, and all patients underwent plain film of the abdomen (KUB) and ultrasound examination (USG) 7 days after the operation to evaluate the stone-free rate. If KUB and USG showed different results, a CT scan was performed. One month after the operation, follow-up was planned, and a double-J stent was removed in the outpatient clinic.

### Statistical analysis

Continuous data were recorded as the mean ± SD if normally distributed or the mean rank if not normally distributed. The student’s t test was used for normally distributed data, and non-normally distributed data were analyzed with the Wilcoxon rank sum test. Chi square tests or Fisher exact tests (proportions) were used for categorical data, and results with *P* < 0.05 was considered to be statistically significant. Statistical analysis was performed using SPSS 23.0 for Windows (SPSS Inc., Chicago, IL).

### Ethical statement

All subjects gave their informed consent for inclusion before they participated in the study. The study was conducted in accordance with the Declaration of Helsinki, and the protocol was approved by the local ethical committee of Tangdu Hospital (TDLL-2015133).

## Results

### Patient characteristics

Ninety patients with complex renal calculi successfully completed surgery. In the 3D printing group, 45 cases, including 25 men and 20 women, were included, and their median age was 45 (23–68) years, including 10 cases of staghorn calculi, 34 cases of multiple renal calculi, and 1 case of horseshoe kidney calculi. 23 cases of left kidney calculi and 22 cases of right kidney calculi were identified. The average diameter of the calculi was 3.81 ± 1.31 cm. There were 45 patients in the control group, including 29 men and 16 women, with an median age of 46 (24–67) years old, including 11 cases of staghorn calculi, 32 cases of multiple renal calculi, 2 cases of horseshoe kidney calculi, 24 cases of left kidney stones, and 21 cases of right kidney stones. The average diameter of the calculi was 3.60 ± 1.35 cm. There was no significant difference in the general data between the two groups (Table 1).

**Table 1:** General information of include patients.

	3D printing group (n = 45)	Control group (n = 45)	*P*
Gender (male/female)	25/20	26/19	0.83
Age (years)	45 (23–68)	46 (24–67)	0.74
Surgical site (left/right)	23/22	24/21	0.83
Staghorn calculi	10	11	0.80
Multiple renal calculi	34	32	0.63
Horseshoe kidneys	1	2	0.56
Diameter of stone (cm)	3.81 ± 1.31	3.60 ± 1.35	0.50
CT value (HU)	930.50 ± 221.73	946.27 ± 257.64	0.76
BMI (kg/m^2^)	24.31 ± 3.42	23.13 ± 3.15	0.09
Hypertension	15.56% (7/45)	13.33% (6/45)	0.76
Diabetes	6.67 (3/45)	4.44% (2/45)	0.65
Creatinine (µmol/L)	80.31 ± 22.01	78.52 ± 18.36	0.68

### Comparison of surgical conditions between the two groups

The overall score of doctor-patient communication (patients or their authorized persons) in the 3D printing group for preoperative communication was 19.32 ± 1.57 points, and that in the control group was 14.51 ± 2.13 points. The score of the 3D printing group was higher than that of the control group, and the difference was statistically significant (*P* < 0.05, 95% CI [4.04–5.58]). An individualized 3D printing model is thus helpful to improve the understanding and satisfaction of doctor-patient communication.

Surgery was successfully completed in all patients in both the 3D printing group and the control group. Among the 45 patients in the 3D printing group, 36 patients underwent surgery by a single channel, and 9 patients had two channels, among which 5 patients underwent punctures in one channel each for the upper calyces and the middle calyces; and 4 patients underwent punctures in one channel each for the upper calyces and the lower calyces. In the control group of 45 patients, 30 cases were treated with a single channel and 15 cases with two channels, among which 7 cases were punctures in one channel each for the upper calyces and the middle calyces; 8 patients were punctures in one channel each for the upper calyces and the lower calyces; and the difference between the two groups was statistically significant. (*P* < 0.05, 95% CI [0.11–0.89]).

In the 3D printing group, the coincidence rate of puncture target calyces with preoperative prediction was 97.78%, and the coincidence rate of the control group was 78.78.0%. The difference between the two groups was statistically significant (*P* < 0.05, 95% CI [1.53–102.97]). The average puncture time of each target calicle of 3D printing group was 7.21 ± 2.32 min, and that of the control group was 9.73 ± 3.61 min. The difference between the two groups was statistically significant (*P* < 0.05, 95% CI [−3.77 to −1.27]). Also, the operation time of the 3D printing group was 103.21 ± 13.49 min, and that of the control group was 126.12 ± 25.87 min. The difference between the two groups was statistically significant (*P* < 0.05, 95% CI [−31.43 to −14.39]). 7 days after the operation, 1 patient in 3D printing group and 2 patients in control group. KUB showed no residual calculi in kidney, USG indicated residual calculi, and CT examination confirmed residual calculi. The calculus clearance rate of the 3D printing group was 96%, while that of the control group was 80%, and the primary calculus clearance rate of the 3D printing group was significantly higher than that of the control group (*P* < 0.05, 95% CI [1.09–26.49]). The length of hospital stay was 6.53 ± 1.36 days in the 3D printing group and 7.31 ± 1.32 days in the control group, and there was a statistically significant difference between the two groups (*P* < 0.05, 95% CI [−1.33 to −0.23]) (Table 2).

**Table 2:** Comparison of surgical conditions between the two groups.

	3D printing group (*n* = 45)	Control group (n = 45)	*P*
Communication score	19.32 ± 1.57	14.51 ± 2.13	< 0.00
Operation time (min)	103.21 ± 13.49	126.12 ± 25.87	< 0.00
Number of channels (1/2)	39/6	30/15	0.03
Puncture coincidence rate	44/45	35/45	0.00
Puncture time (min)	7.21 ± 2.32	9.73 ± 3.61	< 0.00
Clearance rate (%)	96 (43/45)	80 (36/45)	0.03
Drop in hemoglobin (g/L)	6.49 ± 4.52	10.33 ± 9.65	0.02
Complication Clavien I–II Clavien IIIa	3	10	0.02
	0	1	
Hospital stay (D)	6.53 ± 1.36	7.31 ± 1.32	0.00

Postoperative complications were reported based on Clavien Dindo classification. The incidence of postoperative complications was 6.67% in the 3D printing group and 22.22% in the control group. In the 3D printing group, there were one patient in Grade I complication, and the patient developed a perirenal hematoma which was spontaneously resolved. Two patients who with fever (T > 38.5 ℃ or continuous fever for more than 3 days) in Grade II complication, and they had been treated by antibiotic change. There were no Grade III complications in 3D printing group. In the control group, there were two patients in Grade I complication, the two patients developed a perirenal hematoma, and they were spontaneously resolved. There were six patients of Grade II complication in control group, four of them with fever (T > 38.5 ℃ or continuous fever for more than 3 days) and two patients developed pleurisy, and they all had been treated by antibiotic change. One patient with Grade IIIa complication in control group, the patient underwent renal artery embolization due to postoperative bleeding. The complications and decrease in hemoglobin in the 3D printing group were lower than those in the control group (P < 0.05, 95% CI [0.08–0.81]; 95% CI [6.95–0.73]) (Table 3).

**Table 3:** Postoperative complications between the two groups.

	3D printing group (n = 45)	Control group (n = 45)	P
Fever	2	4	
Bleeding	0	1	
Perirenal hematoma	1	2	
Septicopyemia	0	2	
Pleurisy	0	2	
Total	3	11	0.02

## Discussion

Kidney calculus is one of the most common diseases of the urinary system, accounting for 40% of urological surgeries^12^. PCNL has become the gold standard for the treatment of staghorn calculi, multiple renal calculi or lower calyceal calculi due to its advantages of less trauma, less bleeding, a high stone clearance rate and protection of renal tissue. Currently, more than 90% of renal calculi can be treated by PCNL minimally invasive surgery^13, 14^. However, PCNL still has the possibility of serious complications, including intraoperative and postoperative bleeding, which are caused by renal resection, liver, pleural, intestinal injury and other complications^6, 7^. The key critical and difficult point of a PCNL operation is creating the channel for percutaneous nephrolithotomy. The risk of the operation and the rate of calculus clearance are strongly related to the selection and establishment of the percutaneous renal puncture route^15, 16^. Therefore, it a critical point in PCNL surgery has become to clarifying the relationship between the structure of the renal collection system, the distribution of blood vessels and the position of the calculus, to rationally plan the preoperative puncture of the renal calyx and to optimize the operation the steps of the lithotripsy operation. Traditional ultrasound or X-ray guidance can only provide a two-dimensional and incomplete anatomical tissue image^17^. A lack of understanding of the complex three-dimensional morphology of the kidney, unreasonable puncture channel design, and inaccurate puncture of the target calices are the primary causes of renal complications^18^.

3D printing technology converts two-dimensional images into specified materials for printing using computer design software^19–21^. With the concept of biological manufacturing, the application of 3D printing technology in medicine has received increasing attention from researchers worldwide. To date, marked progress has been made in describing the anatomical details of human organs using 3D printing and was first used in preoperative planning and surgical simulation of complex operations^22, 23^. 3D printing has been more commonly used in orthopedics, stomatology and cranial maxillofacial surgery. Giovinco^24^ applied 3D printing technology in preoperative training of Charcot's foot orthopedic surgery and achieved good results. In the operation to repair an acetabular fracture, a simulated pelvis model of the patient is printed with 3D technology, and plate pre-bending, screw length measurement and screw entry direction design are performed on the model, which markedly reduces operation time and surgical complications, and allows for the fracture model to be used for training new doctors^25–28^. 3D printing technology has been applied in percutaneous nephrolithotomy, which can print individual 3D solid models according to the shape of the kidney, calculus and renal blood vessels, and shows the relationship between the kidney and adjacent organs^10^.

To use 3D printing technology with percutaneous nephrolithotomy, Golab A^29^ first used 3D printing technology as surgical guidance for horseshoe kidneys with kidney stones and reported taking 3 min to create a puncture channel. Atalay HA and Brehmer et al.^9, 10^ proposed using three-dimensional reconstructed images (3D-CT) to guide the choice of the puncture location before surgery. This model could effectively improve the understanding of renal anatomy, kidney stone location, surgical procedures, and complications related to surgery and improve patient satisfaction. However, their technique only offers an abstract 3D perspective without a real working model. To use 3D models for preoperative training, xu et al.^11^ reported the use of 3D-printed models combined with percutaneous nephrolithotomy in the treatment of staghorn stones, which suggests that 3D-printed models can potentially be used for preoperative planning in the treatment of full staghorn stones, especially in the selection of the most optimal calyx for puncture. Their studies included a single type of kidney stone, and they did not set up a control group or compare the differences between 3D printing and conventional methods in operation time and complications, thus only showing that this method was feasible. We investigated different types of complex kidney stones, and used a randomized controlled method to compare the difference between 3D printing combined with percutaneous nephroscopy and traditional percutaneous nephroscopy.

In this study, 3D printing technology was used for percutaneous renal channel design, percutaneous renal puncture, intraoperative guidance, doctor-patient communication and other aspects, and its clinical application effect was evaluated. Results of this study showed that the operation time and hospital stay of the 3D printing group were shorter than those of the conventional group; the amount of surgical blood loss and the incidence of complications were lower than those of the conventional group; and the calculus clearance rate and doctor-patient communication were higher than those of the conventional group (*P* < 0.05). These results suggest that 3D printing is a safe and effective method to treat complex renal calculi. The possible reason was 3D printing can objectively and accurately describes the anatomical structure of the kidney, making it easier for patients and guardians to understand the operation process and operation risk. This information exchange may enhance the effectiveness of doctor-patient communication. Through preoperative planning, we designed the best gravel channel that forms an obtuse angle with the surrounding target renal calyces, and it is convenient to deal with stones in different renal calyces. So the stone clearance rate was higher than that of the control group. After Preoperative puncture training the success rate of puncture was improved, the lithotripsy efficiency was improved, the number of channels to be punctured was reduced, and the time to create a channel was shortened. In this study, there is no significant difference in the stones size and general conditions between the two groups before operation. The operation was completed by the same operator. The number of punctures in the 3D printing group was significantly less than that in the control group. Thus, the operation time was shorter than that of the control group. 3D printing can clearly show the distribution of blood vessels in the kidney, the size and location of the calculus, and increase puncture accuracies. With the decrease in puncture channels and the improvement in puncture accuracy, operation time was shortened, kidney injury was reduced, and the amount of blood loss, damage to adjacent organs, infection-related complications were also reduced. These outcomes may be the primary reason why the complication rate of the 3D printing group was significantly lower than that of the control group.

However, there were some limitations to this study. As an investigative study, the sample size might be a bit small to be definitive. We are planning to continue this study and may invite other institutions to participate in this project. Also, as a new technology, the cost of 3D printing for each surgery is approximately 500 US dollars, which increases patient burden. Finally, this model adopts the prone position consistent with PCNL during CT examination. However, the position of PCNL now includes oblique lithotomy position and lateral lying position. Different positions will lead to differences between stones and surrounding tissues. Therefore, in the future, we should adjust the patient's position during CT examination according to the operator's requirements for position, so as to restore the most real data.

## Conclusion

The application of 3D printing technology in complex percutaneous nephrolithotomy can shorten operation time, reduce surgical bleeding, improve stone removal rates, reduce complications, and accelerate patient recovery. Thus, 3D printing is a safe and effective method to treat complex renal calculi. However, with the progression of materials science and engineering technology, the application of 3D printing in percutaneous nephrolithotomy may be further strengthened, but the combination of 3D printing and percutaneous nephrolithotomy in the treatment of kidney stones still requires further research and development.
